# (*E*)-4-Hy­droxy-*N*′-(4-hy­droxy-3-meth­oxy­benzyl­idene)benzohydrazide

**DOI:** 10.1107/S1600536810045162

**Published:** 2010-11-13

**Authors:** Marwan Shalash, Abdussalam Salhin, Rohana Adnan, Chin Sing Yeap, Hoong-Kun Fun

**Affiliations:** a School of Chemical Sciences, Universiti Sains Malaysia, 11800 USM, Penang, Malaysia; b X-ray Crystallography Unit, School of Physics, Universiti Sains Malaysia, 11800 USM, Penang, Malaysia

## Abstract

In the title compound, C_15_H_14_N_2_O_4_, the N=C double bond has an *E* configuration. The two benzene rings make a dihedral angle of 28.59 (6)°. In the crystal, mol­ecules are linked into a three-dimensional network by inter­molecular N—H⋯O, O—H⋯O and C—H⋯O hydrogen bonds and stabilized by weak C—H⋯π inter­actions.

## Related literature

For the pharmacological activity of Schiff bases derivatives, see: Zia-ur-Rehman *et al.* (2009 ▶); Parashar *et al.* (1988 ▶); Hadjoudis *et al.* (1987 ▶). For the biological activity of hydrazide derivatives, see: Waisser *et al.* (1990 ▶); Hall *et al.* (1993 ▶); Salhin *et al.* (2007 ▶, 2009 ▶); Tameem *et al.* (2006 ▶, 2007 ▶, 2008 ▶). For the stability of the temperature controller used in the data collection, see: Cosier & Glazer (1986 ▶).
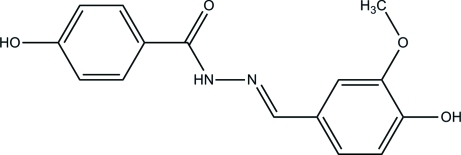


## Experimental

### 

#### Crystal data

C_15_H_14_N_2_O_4_
*M* *_r_* = 286.28
Orthorhombic, 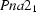

*a* = 10.9034 (3) Å
*b* = 8.5533 (2) Å
*c* = 14.7437 (4) Å
*V* = 1375.00 (6) Å^3^
*Z* = 4
Mo *K*α radiation
μ = 0.10 mm^−1^
*T* = 100 K
0.43 × 0.34 × 0.16 mm

#### Data collection

Bruker SMART APEXII CCD area-detector diffractometer
Absorption correction: multi-scan (*SADABS*; Bruker, 2009 ▶) *T*_min_ = 0.958, *T*_max_ = 0.984
8269 measured reflections
2098 independent reflections
2033 reflections with *I* > 2σ(*I*)
*R*_int_ = 0.021

#### Refinement

*R*[*F* ^2^ > 2σ(*F* ^2^)] = 0.033
*wR*(*F* ^2^) = 0.087
*S* = 1.03
2098 reflections
203 parameters
1 restraint
H atoms treated by a mixture of independent and constrained refinement
Δρ_max_ = 0.35 e Å^−3^
Δρ_min_ = −0.33 e Å^−3^

### 

Data collection: *APEX2* (Bruker, 2009 ▶); cell refinement: *SAINT* (Bruker, 2009 ▶); data reduction: *SAINT*; program(s) used to solve structure: *SHELXTL* (Sheldrick, 2008 ▶); program(s) used to refine structure: *SHELXTL*; molecular graphics: *SHELXTL*; software used to prepare material for publication: *SHELXTL* and *PLATON* (Spek, 2009 ▶).

## Supplementary Material

Crystal structure: contains datablocks global, I. DOI: 10.1107/S1600536810045162/fj2361sup1.cif

Structure factors: contains datablocks I. DOI: 10.1107/S1600536810045162/fj2361Isup2.hkl

Additional supplementary materials:  crystallographic information; 3D view; checkCIF report

## Figures and Tables

**Table 1:** Hydrogen-bond geometry (Å, °) *Cg*1 is the centroid of the C1–C6 benzene ring.

*D*—H⋯*A*	*D*—H	H⋯*A*	*D*⋯*A*	*D*—H⋯*A*
N1—H1*N*1⋯O3^i^	0.84 (2)	2.18 (2)	2.9534 (16)	153.0 (17)
N1—H1*N*1⋯O4^i^	0.84 (2)	2.53 (2)	3.2252 (16)	140.0 (17)
O1—H1*O*1⋯O2^ii^	0.81 (3)	1.84 (3)	2.6361 (15)	165 (2)
O4—H1*O*4⋯O1^iii^	0.87 (2)	1.89 (2)	2.7457 (16)	170 (3)
C2—H2*A*⋯O2^ii^	0.93	2.45	3.1271 (17)	129
C15—H15*B*⋯*Cg*1^iv^	0.96	2.81	3.5620 (14)	132

Symmetry codes: (i) 
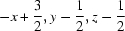
; (ii) 
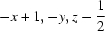
; (iii) 
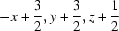
; (iv) 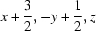.

**Fig. 1.**
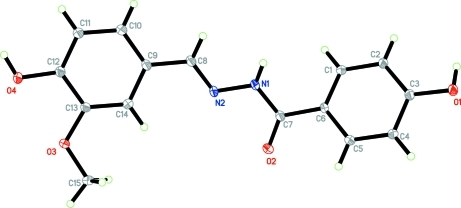
The molecular structure of title compound with atom labels and 50% probability ellipsoids for non-H atoms.

**Fig. 2.**
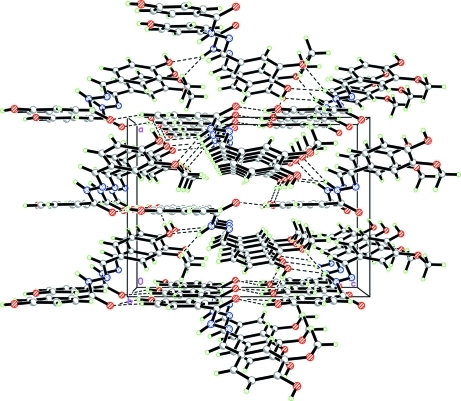
The crystal packing of title compound viewed down b axis, showing the molecules are linked into a three-dimensional network.

## supplementary crystallographic information

### Comment

Syntheses based on Schiff bases have become a major attraction in Chemistry because these products are well known for their pharmacological properties such as anti-tumor, anti-bacterial, anti-oxidant (Zia-ur-Rehman *et al.*, 2009; Parashar *et al.*, 1988) and photochromic activities (Hadjoudis *et al.*, 1987). Many hydrazide derivatives known to have significant biological activities such as monoamine oxidase inhibitory activity, antifungal and tuberculostatic activity (Waisser *et al.*, 1990; Hall *et al.*, 1993). Continuing our interest on the synthesis and application of hydrazone and hydrazide derivatives (Salhin *et al.*, 2007, 2009; Tameem *et al.*, 2006, 2007, 2008), compound (I) (Fig. 1) was hereby synthesized based on Schiff bases by the condensation reaction of 4-hydroxybenzhydrazide and 4-hydroxy-3-methoxybenzaldehyde. The crystal structure is presented here.

The N═C double bond of (I) exist in an *E*-configuration. The two benzene rings make dihedral angle of 28.59 (6)°. The methoxy group is almost planar with its attached benzene ring [torsion angle 6.3 (2)°]. In the crystal packing, the molecules are linked into a three-dimensional network by intermolecular N—H···O, O—H···O and C—H···O hydrogen bonds and stabilized by weak C—H···π interactions (Fig. 2, Table 1).

### Experimental

A mixture of 4-hydroxybenzhydrazide (0.2 g, 1.31 mmol) and 4-hydroxy-3-methoxybenzaldehyde (0.2 g, 1.31 mmol) in 30 ml of methanol containing few drops of acetic acid was refluxed for about 5 h. On cooling to room temperature, a solid precipitate was formed. The solid was filtered and then recrystallized from ethanol. Yellow crystals of (I) suitable for X-ray diffraction were obtained by slow evaporation of solution.

### Refinement

The O– and N-bound hydrogen atoms were located from difference Fourier map and refined freely. The rest of hydrogen atoms were positioned geometrically [C–H = 0.93 & 0.96 Å] and refined using a riding model [*U*_iso_(H) = 1.2 & 1.5*U*_eq_(C)]. A rotating-group model were applied for methyl groups. 1538 Friedel pairs were merged before final refinement. The absolute configuration is unknown.

### Crystal data

**Table:**

C_15_H_14_N_2_O_4_	*F*(000) = 600
*M**_r_* = 286.28	*D*_x_ = 1.383 Mg m^−^^3^
Orthorhombic, *P**n**a*2_1_	Mo *K*α radiation, λ = 0.71073 Å
Hall symbol: P 2c -2n	Cell parameters from 5243 reflections
*a* = 10.9034 (3) Å	θ = 2.8–30.1°
*b* = 8.5533 (2) Å	µ = 0.10 mm^−^^1^
*c* = 14.7437 (4) Å	*T* = 100 K
*V* = 1375.00 (6) Å^3^	Plate, yellow
*Z* = 4	0.43 × 0.34 × 0.16 mm

### Data collection

**Table:**

Bruker SMART APEXII CCD area-detector diffractometer	2098 independent reflections
Radiation source: fine-focus sealed tube	2033 reflections with *I* > 2σ(*I*)
graphite	*R*_int_ = 0.021
φ and ω scans	θ_max_ = 30.1°, θ_min_ = 2.8°
Absorption correction: multi-scan (*SADABS*; Bruker, 2009)	*h* = −12→15
*T*_min_ = 0.958, *T*_max_ = 0.984	*k* = −12→8
8269 measured reflections	*l* = −18→20

### Refinement

**Table:**

Refinement on *F*^2^	Primary atom site location: structure-invariant direct methods
Least-squares matrix: full	Secondary atom site location: difference Fourier map
*R*[*F*^2^ > 2σ(*F*^2^)] = 0.033	Hydrogen site location: inferred from neighbouring sites
*wR*(*F*^2^) = 0.087	H atoms treated by a mixture of independent and constrained refinement
*S* = 1.03	*w* = 1/[σ^2^(*F*_o_^2^) + (0.0687*P*)^2^ + 0.0626*P*] where *P* = (*F*_o_^2^ + 2*F*_c_^2^)/3
2098 reflections	(Δ/σ)_max_ < 0.001
203 parameters	Δρ_max_ = 0.35 e Å^−^^3^
1 restraint	Δρ_min_ = −0.32 e Å^−^^3^

### Special details

**Table:**

Experimental. The crystal was placed in the cold stream of an Oxford Cryosystems Cobra open-flow nitrogen cryostat (Cosier & Glazer, 1986) operating at 100.0 (1) K.
Geometry. All e.s.d.'s (except the e.s.d. in the dihedral angle between two l.s. planes) are estimated using the full covariance matrix. The cell e.s.d.'s are taken into account individually in the estimation of e.s.d.'s in distances, angles and torsion angles; correlations between e.s.d.'s in cell parameters are only used when they are defined by crystal symmetry. An approximate (isotropic) treatment of cell e.s.d.'s is used for estimating e.s.d.'s involving l.s. planes.
Refinement. Refinement of *F*^2^ against ALL reflections. The weighted *R*-factor *wR* and goodness of fit *S* are based on *F*^2^, conventional *R*-factors *R* are based on *F*, with *F* set to zero for negative *F*^2^. The threshold expression of *F*^2^ > σ(*F*^2^) is used only for calculating *R*-factors(gt) *etc*. and is not relevant to the choice of reflections for refinement. *R*-factors based on *F*^2^ are statistically about twice as large as those based on *F*, and *R*- factors based on ALL data will be even larger.

### Fractional atomic coordinates and isotropic or equivalent isotropic displacement parameters (Å^2^)

**Table:**

	*x*	*y*	*z*	*U*_iso_*/*U*_eq_	
O1	0.50848 (11)	−0.29559 (13)	−0.39327 (7)	0.0176 (2)	
O2	0.47208 (11)	0.16057 (13)	−0.05335 (7)	0.0185 (2)	
O3	0.72455 (10)	0.71644 (12)	0.21457 (7)	0.0135 (2)	
O4	0.84067 (11)	0.96468 (12)	0.16052 (7)	0.0172 (2)	
N1	0.59306 (12)	0.30690 (14)	−0.14694 (8)	0.0134 (2)	
N2	0.61084 (12)	0.41786 (14)	−0.07972 (8)	0.0130 (2)	
C1	0.51462 (13)	0.09777 (17)	−0.29247 (9)	0.0122 (3)	
H1A	0.5163	0.2022	−0.3099	0.015*	
C2	0.51039 (13)	−0.01823 (17)	−0.35842 (9)	0.0131 (3)	
H2A	0.5080	0.0083	−0.4196	0.016*	
C3	0.50984 (13)	−0.17538 (18)	−0.33189 (10)	0.0133 (3)	
C4	0.51091 (14)	−0.21469 (17)	−0.23966 (9)	0.0149 (3)	
H4A	0.5106	−0.3191	−0.2221	0.018*	
C5	0.51242 (13)	−0.09687 (17)	−0.17432 (9)	0.0138 (3)	
H5A	0.5107	−0.1230	−0.1131	0.017*	
C6	0.51643 (13)	0.05963 (16)	−0.20017 (9)	0.0115 (3)	
C7	0.52375 (13)	0.17934 (17)	−0.12730 (9)	0.0127 (3)	
C8	0.68511 (14)	0.52860 (17)	−0.10081 (9)	0.0133 (3)	
H8A	0.7178	0.5333	−0.1590	0.016*	
C9	0.71871 (13)	0.64765 (17)	−0.03369 (9)	0.0127 (3)	
C10	0.78103 (14)	0.78203 (17)	−0.06151 (10)	0.0148 (3)	
H10A	0.7962	0.7984	−0.1228	0.018*	
C11	0.82067 (14)	0.89195 (17)	0.00196 (10)	0.0153 (3)	
H11A	0.8615	0.9815	−0.0172	0.018*	
C12	0.79915 (14)	0.86768 (16)	0.09385 (9)	0.0130 (3)	
C13	0.73610 (12)	0.73180 (16)	0.12227 (9)	0.0114 (3)	
C14	0.69530 (14)	0.62378 (17)	0.05952 (10)	0.0127 (3)	
H14A	0.6526	0.5356	0.0786	0.015*	
C15	0.67731 (14)	0.57037 (17)	0.24666 (10)	0.0159 (3)	
H15A	0.6747	0.5713	0.3117	0.024*	
H15B	0.7295	0.4869	0.2265	0.024*	
H15C	0.5961	0.5549	0.2232	0.024*	
H1N1	0.638 (2)	0.311 (2)	−0.1935 (17)	0.018 (5)*	
H1O1	0.501 (2)	−0.252 (3)	−0.442 (2)	0.043 (8)*	
H1O4	0.890 (2)	1.034 (3)	0.138 (2)	0.033 (6)*	

### Atomic displacement parameters (Å^2^)

**Table:**

	*U*^11^	*U*^22^	*U*^33^	*U*^12^	*U*^13^	*U*^23^
O1	0.0328 (6)	0.0102 (5)	0.0099 (4)	0.0016 (4)	−0.0035 (4)	−0.0002 (4)
O2	0.0279 (6)	0.0167 (5)	0.0109 (4)	−0.0050 (4)	0.0046 (4)	−0.0017 (4)
O3	0.0187 (5)	0.0131 (5)	0.0088 (4)	−0.0029 (4)	0.0002 (4)	0.0007 (3)
O4	0.0272 (6)	0.0118 (5)	0.0125 (4)	−0.0068 (4)	−0.0022 (4)	−0.0013 (4)
N1	0.0181 (5)	0.0127 (5)	0.0093 (5)	−0.0021 (4)	0.0027 (4)	−0.0035 (4)
N2	0.0181 (6)	0.0111 (5)	0.0099 (5)	−0.0010 (4)	−0.0003 (4)	−0.0031 (4)
C1	0.0153 (6)	0.0109 (6)	0.0104 (5)	−0.0005 (5)	−0.0001 (5)	0.0011 (5)
C2	0.0182 (7)	0.0101 (6)	0.0109 (6)	−0.0001 (5)	−0.0016 (5)	0.0000 (4)
C3	0.0178 (6)	0.0114 (6)	0.0109 (6)	0.0002 (5)	−0.0012 (5)	−0.0009 (5)
C4	0.0228 (7)	0.0106 (6)	0.0111 (6)	−0.0009 (5)	−0.0013 (5)	0.0014 (5)
C5	0.0190 (7)	0.0127 (7)	0.0096 (5)	−0.0011 (5)	−0.0010 (5)	0.0005 (5)
C6	0.0142 (6)	0.0109 (6)	0.0095 (5)	−0.0012 (5)	0.0001 (5)	−0.0019 (5)
C7	0.0159 (6)	0.0120 (6)	0.0104 (6)	0.0003 (5)	−0.0003 (5)	−0.0014 (5)
C8	0.0165 (7)	0.0137 (6)	0.0099 (5)	0.0002 (5)	0.0003 (5)	−0.0017 (4)
C9	0.0152 (6)	0.0127 (6)	0.0101 (5)	−0.0003 (5)	−0.0007 (4)	−0.0015 (4)
C10	0.0192 (7)	0.0141 (6)	0.0112 (5)	−0.0019 (5)	0.0010 (5)	−0.0003 (5)
C11	0.0212 (7)	0.0122 (6)	0.0125 (6)	−0.0026 (5)	0.0003 (5)	0.0006 (5)
C12	0.0174 (6)	0.0104 (6)	0.0111 (5)	−0.0001 (5)	−0.0008 (5)	−0.0008 (5)
C13	0.0135 (6)	0.0110 (6)	0.0099 (6)	0.0003 (5)	−0.0003 (5)	0.0008 (4)
C14	0.0142 (6)	0.0117 (6)	0.0122 (5)	−0.0015 (5)	0.0000 (5)	0.0004 (5)
C15	0.0211 (7)	0.0145 (6)	0.0119 (6)	−0.0026 (5)	0.0010 (5)	0.0030 (5)

### Geometric parameters (Å, °)

**Table:**

O1—C3	1.3698 (17)	C4—H4A	0.9300
O1—H1O1	0.82 (3)	C5—C6	1.393 (2)
O2—C7	1.2377 (18)	C5—H5A	0.9300
O3—C13	1.3729 (16)	C6—C7	1.4862 (18)
O3—C15	1.4318 (17)	C8—C9	1.4664 (18)
O4—C12	1.3637 (17)	C8—H8A	0.9300
O4—H1O4	0.86 (3)	C9—C10	1.397 (2)
N1—C7	1.3584 (19)	C9—C14	1.4127 (18)
N1—N2	1.3859 (15)	C10—C11	1.395 (2)
N1—H1N1	0.84 (2)	C10—H10A	0.9300
N2—C8	1.2844 (19)	C11—C12	1.3905 (19)
C1—C2	1.3900 (19)	C11—H11A	0.9300
C1—C6	1.3995 (19)	C12—C13	1.4139 (19)
C1—H1A	0.9300	C13—C14	1.3811 (19)
C2—C3	1.400 (2)	C14—H14A	0.9300
C2—H2A	0.9300	C15—H15A	0.9600
C3—C4	1.4008 (19)	C15—H15B	0.9600
C4—C5	1.3943 (19)	C15—H15C	0.9600
			
C3—O1—H1O1	104 (2)	N2—C8—C9	120.42 (12)
C13—O3—C15	116.36 (11)	N2—C8—H8A	119.8
C12—O4—H1O4	110.3 (18)	C9—C8—H8A	119.8
C7—N1—N2	118.39 (11)	C10—C9—C14	119.50 (12)
C7—N1—H1N1	122.1 (14)	C10—C9—C8	119.66 (12)
N2—N1—H1N1	118.1 (14)	C14—C9—C8	120.73 (12)
C8—N2—N1	114.84 (11)	C11—C10—C9	120.55 (13)
C2—C1—C6	120.95 (13)	C11—C10—H10A	119.7
C2—C1—H1A	119.5	C9—C10—H10A	119.7
C6—C1—H1A	119.5	C12—C11—C10	120.04 (14)
C1—C2—C3	119.35 (13)	C12—C11—H11A	120.0
C1—C2—H2A	120.3	C10—C11—H11A	120.0
C3—C2—H2A	120.3	O4—C12—C11	123.74 (13)
O1—C3—C2	122.42 (13)	O4—C12—C13	116.61 (12)
O1—C3—C4	117.47 (13)	C11—C12—C13	119.57 (13)
C2—C3—C4	120.11 (13)	O3—C13—C14	124.78 (12)
C5—C4—C3	119.82 (14)	O3—C13—C12	114.65 (12)
C5—C4—H4A	120.1	C14—C13—C12	120.53 (12)
C3—C4—H4A	120.1	C13—C14—C9	119.79 (13)
C6—C5—C4	120.40 (13)	C13—C14—H14A	120.1
C6—C5—H5A	119.8	C9—C14—H14A	120.1
C4—C5—H5A	119.8	O3—C15—H15A	109.5
C5—C6—C1	119.33 (13)	O3—C15—H15B	109.5
C5—C6—C7	117.78 (12)	H15A—C15—H15B	109.5
C1—C6—C7	122.89 (13)	O3—C15—H15C	109.5
O2—C7—N1	123.04 (13)	H15A—C15—H15C	109.5
O2—C7—C6	121.53 (13)	H15B—C15—H15C	109.5
N1—C7—C6	115.41 (12)		
			
C7—N1—N2—C8	174.70 (13)	N2—C8—C9—C10	−168.42 (14)
C6—C1—C2—C3	−1.0 (2)	N2—C8—C9—C14	15.4 (2)
C1—C2—C3—O1	−178.53 (13)	C14—C9—C10—C11	0.3 (2)
C1—C2—C3—C4	1.3 (2)	C8—C9—C10—C11	−175.92 (14)
O1—C3—C4—C5	179.95 (13)	C9—C10—C11—C12	0.5 (2)
C2—C3—C4—C5	0.1 (2)	C10—C11—C12—O4	176.23 (14)
C3—C4—C5—C6	−1.9 (2)	C10—C11—C12—C13	−0.5 (2)
C4—C5—C6—C1	2.2 (2)	C15—O3—C13—C14	6.3 (2)
C4—C5—C6—C7	−177.19 (13)	C15—O3—C13—C12	−171.28 (12)
C2—C1—C6—C5	−0.8 (2)	O4—C12—C13—O3	0.37 (18)
C2—C1—C6—C7	178.59 (13)	C11—C12—C13—O3	177.34 (14)
N2—N1—C7—O2	2.8 (2)	O4—C12—C13—C14	−177.32 (13)
N2—N1—C7—C6	−175.54 (12)	C11—C12—C13—C14	−0.3 (2)
C5—C6—C7—O2	−34.2 (2)	O3—C13—C14—C9	−176.25 (13)
C1—C6—C7—O2	146.44 (15)	C12—C13—C14—C9	1.2 (2)
C5—C6—C7—N1	144.17 (13)	C10—C9—C14—C13	−1.2 (2)
C1—C6—C7—N1	−35.2 (2)	C8—C9—C14—C13	175.02 (13)
N1—N2—C8—C9	−175.73 (12)		

### Hydrogen-bond geometry (Å, °)

**Table:**

Cg1 is the centroid of the C1–C6 benzene ring.

**Table:**

*D*—H···*A*	*D*—H	H···*A*	*D*···*A*	*D*—H···*A*
N1—H1N1···O3^i^	0.84 (2)	2.18 (2)	2.9534 (16)	153.0 (17)
N1—H1N1···O4^i^	0.84 (2)	2.53 (2)	3.2252 (16)	140.0 (17)
O1—H1O1···O2^ii^	0.81 (3)	1.84 (3)	2.6361 (15)	165 (2)
O4—H1O4···O1^iii^	0.87 (2)	1.89 (2)	2.7457 (16)	170 (3)
C2—H2A···O2^ii^	0.93	2.45	3.1271 (17)	129
C15—H15B···Cg1^iv^	0.96	2.81	3.5620 (14)	132

Symmetry codes: (i) −*x*+3/2, *y*−1/2, *z*−1/2; (ii) −*x*+1, −*y*, *z*−1/2; (iii) −*x*+3/2, *y*+3/2, *z*+1/2; (iv) *x*+3/2, −*y*+1/2, *z*.
